# Extension of Lecture Terms

**Published:** 1848-05

**Authors:** 


*Extension of Lecture Terms.*—The last number of the Southern Medical and Surgical Journal contains a statement, which, in justice to the Medical College of Georgia, should be generally known. It is, that the Georgia College in 1832 was organized upon a six months course of Lectures, and for five successive sessions, this plan was continued. Finding that the classes did not increase, in 1835 a circular was addressed to every medical College in the United States, proposing the adoption of the same length of term. No other institution, however, acceeding to the proposition, the Georgia school reluctantly shortened its term to four months. We take pleasure in aiding to give currency to the foregoing statement, in order that the merit of priority in the matter may be attached to the institution to which it rightfully belongs.

